# Investigation on the Microstructure and Wear Behavior of Laser-Cladded High Aluminum and Chromium Fe-B-C Coating

**DOI:** 10.3390/ma13112443

**Published:** 2020-05-27

**Authors:** Jingjing Li, Jiang Ju, Weiwei Chang, Chao Yang, Jun Wang

**Affiliations:** 1 School of Materials Science and Engineering, Shanghai Jiao Tong University, Shanghai 200240, China; lijingjing-41422134@sjtu.edu.cn (J.L.); jujiang1990@sjtu.edu.cn (J.J.); yangchao1987@sjtu.edu.cn (C.Y.); 2 Shanghai Key Laboratory of Advanced High-temperature Materials and Precision Forming, Shanghai Jiao Tong University, Shanghai 200240, China; 3 Institute for Advanced Materials and Technology, University of Science and Technology Beijing, Beijing 100083, China; s20181310@xs.ustb.edu.cn

**Keywords:** Fe-based coating, laser cladding, transmission electron microscopy (TEM), microstructure, wear resistance

## Abstract

In this study, a high aluminum and chromium Fe-B-C coating was prepared using laser cladding on 2Cr13 steel substrate. The microstructure, microhardness, and wear resistance of the high aluminum and chromium Fe-B-C coating were investigated. The results show that this dense coating possesses good metallurgical bond with the substrate. The microstructure is mainly composed of α-(Fe, Cr, Al) lath martensite, orthorhombic M_2_B boride, orthogonal M_3_C_2_, and orthorhombic M_7_C_3_ carbides. The microhardness of the coating can reach 620 HV which is 3.3-times higher than that (190 HV) of the substrate. The coating shows a lower friction coefficient of 0.75 than that of the substrate (1.08). The wear rates of the substrate and the coating are 0.295 mg/min and 0.103 mg/min, respectively, indicating the coating exhibits excellent wear resistance. The wear mechanism transforms severe adhesive wear and abrasive wear of the substrate to slight abrasive wear of the coating. The results can provide technical support to improve the properties of the Fe-based laser cladded coating.

## 1. Introduction

With the rapid development of the world economy, global energy demand is steadily on the increase [1,2]. The non-renewable fossil fuels accounts for more than 80% of the total energy, which puts forward higher request to the mining equipment [3,4]. The wear of the metal parts is one of the main problems affecting the service life of mining equipment. Thus, it is urgent and crucial to improve the wear resistance of mining equipment [5].

Laser cladding technology is a material surface modification method for improving the wear resistance of alloys [6]. It can fabricate a coating with special physical, chemical, or mechanical properties using a high energy laser beam [7]. Compared with traditional spraying processes, the laser cladding technology possesses four advantages: (1) Good metallurgical bonding with low dilution; (2) High yield and small margin for subsequent processing; (3) precise control, online control and remote manipulation for the repair of local and critical parts; and (4) low cost [8,9,10].

The coatings for laser cladding mainly consist of Fe-based, Co-based, Ni-based, ceramics, etc. [11]. Co-based and Ni-based coatings are characterized by high hardness, excellent wear resistance, heat resistance, and oxidation resistance. However, the cost of Co-based and Ni-based alloys is very high [12,13]. Ceramic coatings have much better properties in comparison with metal coatings, but its brittleness has always been an obstacle to its wide application [14]. Hence, Fe-based coatings attract more and more attention due to their low cost and high performance [15]. Many scholars have studied the improvement of wear resistance of materials by Fe-based coatings via laser cladding [16]. W.J. Wang et al. fabricated Fe-Cr-Ni-B composite coating with high hardness and good wear resistance on wheel and rail materials using laser cladding [17]. Zhang et al. studied the microstructure, wear resistance and corrosion resistance of Fe-Ti-V-Cr-C-CeO_2_ coating, and found the coating possessed good wear and corrosion resistance [18].

H.G. Fu et al. found the addition of Al and Cr can significantly enhance the microhardness, wear resistance and oxidation resistance Fe-B coatings [19,20,21]. In this study, the high aluminum and chromium Fe-B-C coating was fabricated using laser cladding technology on the 2Cr23 steel substrate. The microstructure, microhardness and wear resistance of the high aluminum and chromium Fe-B-C coating was investigated.

## 2. Materials Fabrication and Experimental Methods

### 2.1. Materials Fabrication

**Figure 1:**
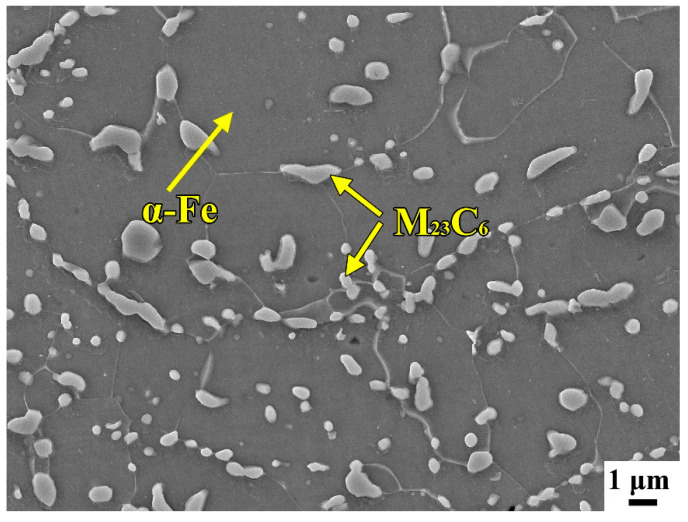
The microstructure image of the 2Cr13 steel substrate.

The 2Cr13 steel (corresponding to 420 steel, US ASTM Standards) with a dimension of 150 mm × 150 mm × 10 mm was used to the substrate, its chemical compositions are 0.22 wt.% C, 12.9 wt.% Cr, 0.4 wt.% Si, 0,61 wt.% Mn, 0.016 wt.% S, 0.018 P wt.%, and the balance Fe. The microstructure of the 2Cr13 substrate is mainly composed of the α-Fe matrix and the M_23_C_6_ carbides distributed along the grain boundaries, as shown in Figure 1. The 2Cr13 substrate was grinded using the 1000# SiC sandpaper, and then cleaned by alcohol and acetone. High aluminum and chromium Fe-B-C powder was proportioned with pure alloy powder with a size of 60–80 μm. The prepared high aluminum and chromium Fe-B-C powder was dried in a drying oven to remove moisture, and then put into a ball mill pot in a glove box and ground at 120 r/min for 4 h. The chemical compositions of high aluminum and chromium Fe-B-C powder after mixing were measured by inductively coupled plasma mass spectrometry (ICP-MS), as shown in Table 1.

In this study, IPG-6000 optical fiber laser cladding system was used for laser cladding. First, a layer of high aluminum and chromium Fe-B-C powder with a thickness of 1 mm was prefabricated on 2Cr13 steel plate. The binder was cellulose acetate (C_6_H_8_O_3_ (COOCH_3_)_2_)_n_. Before laser cladding, the 2Cr13 substrate was preheated to 80 °C to reduce cracking. The main parameters are 2.5 kW laser power, 4 mm/s scanning speed, 15 L/min-Ar gas flow speed, and 5 mm × 5 mm spot size. The metallographic sample with a size of 10 mm × 10 mm × 5 mm was prepared by wire cutting. After grinding and polishing, it was cleaned using acetone and alcohol for 60 s.

### 2.2. Phase Diagram Calculation

Thermo-Calc^®^ software was used to calculated the phase diagram. The mass percent of each element was C-0.3 wt.%, Cr-10.0 wt.%, Al-6.0 wt.%, Si-0.6 wt.%, Mn-0.7 wt.%, and the balance Fe. The content of B varied from 0.0 to 3.0 wt.%. The POLY-3 module provided initial conditions for the phase diagram calculations, such as composition, temperature, activity etc. The thermodynamic data for each substance was provided by the TCFE8 database. Furthermore, multivariate and multiphase equilibrium calculations were performed. The temperature range was 0–1750 °C, and the pressure was 105 kPa.

### 2.3. Microhardness and Wear Resistance Tests

The microhardness was measured using the MICRO MET-5103 Digital micrometer. The normal load was 4.9 N, the load time was 10 s. The UMT-3 reciprocating wear tester was conducted to test the wear resistance. The Al_2_O_3_ ceramic ball with Ø 4 mm was the grinding material, its microhardness is 1300 HV [21]. The load was 20 N, the reciprocating stroke was set as 5 mm, the frequency was 1 Hz. The wear time and the sliding speed were 60 min and 0.1 m/s, respectively. The wear resistance was evaluated using wear rate (*Wr*, g^3^·N^−1^·min^−1^) which can be calculated according to the following Equation (1) [22]:

(1) $$W_{r}=\frac{w_{loss}}{F_{n}\times t}$$

where *w_loss_* represents the total wear loss (mg), *F_n_* is the normal load (N) and *t* is the wear time (s). The microhardness and wear tests were repeated three times for each sample to ensure more accurate experimental data.

### 2.4. Characterization

The samples were ground on SiC papers with granulations of 240, 400, 600, 800, 1200, 1500, 2000, and 3000 and polished using diamond suspensions. The samples were etched using 5% FeCl_3_ solution. The microstructure and compositional analyses of various phases were performed using a Mira 3 scanning eletron microscope (SEM), transmission electron microscopy (TEM) and a JXA-8230 electron probe micro-analyzer (EPMA). The EPMA parameters were set to the 15 kV acceleration voltage, 10 nA beam current and 1 μm beam spot diameter. The standard sample was pure material (such as pure Al, Fe etc.). X-ray diffraction (XRD) was performed on a SHIMADZU Japan XRD-7000 diffractometer with copper Kα radiation coupling continuous scanning at 40 kV and 200 mA as the X-ray source. The specimen was scanned in the angular 2θ ranging from 20° to 80° with a step size of 0.2° and a collection time of 10 s.

## 3. Results and Discussions

### 3.1. Phase Diagram and Phase Structure

**Figure 2:**
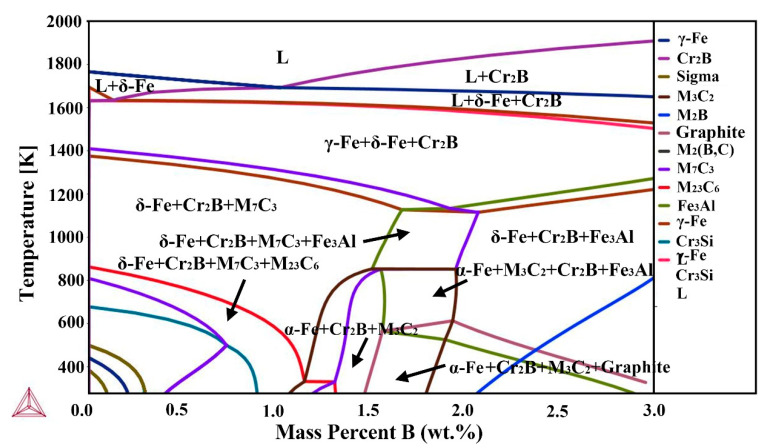
The calculated vertical sections of Fe-Cr-B-Al-C high aluminum and chromium Fe-B-C coatings.

The vertical sections of high aluminum and chromium Fe-B-C coatings were calculated using Themo-Calc® software, as shown in Figure 2. From Figure 2, under the condition of the equilibrium solidification, Cr_2_B phase precipitates directly from the liquid phase when the concentration of B is 1.5 wt.%. The eutectic reaction of L→δ-Fe + Cr_2_B occurs at about 1400 °C, precipitating δ-Fe phase. As the temperature continues to reduce, the remaining liquid phase transform into the γ-Fe, forming the δ-Fe + γ-Fe + Cr_2_B three-phase zone. When the temperature drops to about 900 °C, the M_7_C_3_ phase gradually precipitates. It can be seen that the equilibrium microstructure consists mainly of α-Fe + Cr_2_B + M_3_C_2_ at room temperature. In addition, the M_7_C_3_ and Fe_3_Al phase may be obtained when the B content is less and greater than 1.5 wt.%, which also may be obtained under the rapid cooling.

### 3.2. Microstructure

**Figure 3:**
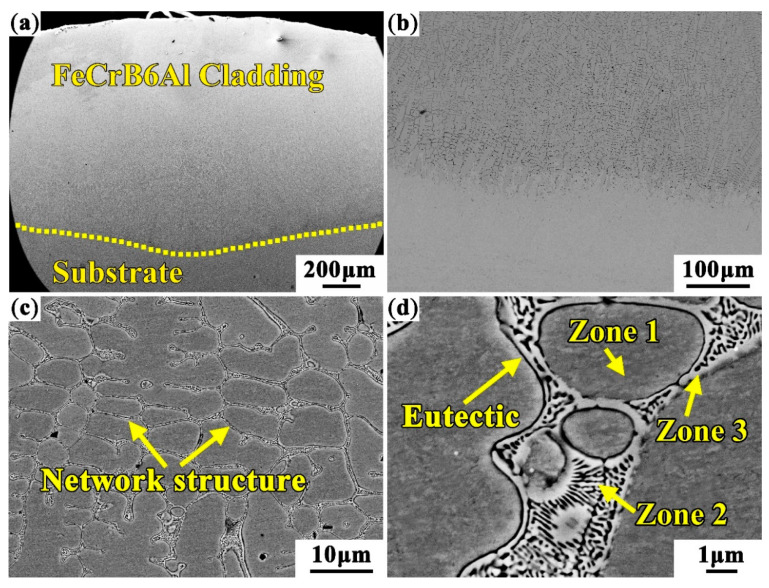
The microstructure morphologies of high aluminum and chromium Fe-B-C coating: (**a**) Macromorphology; (**b**) Bottom of cladding layer; (**c**,**d**) Middle part of cladding layer.

Figure 3 shows the microstructure morphologies of high aluminum and chromium Fe-B-C coating. From Figure 3a, the coating is uniform and dense, and no defects such as pores and cracks are found. Figure 3b shows the coating has a good metallurgical bonding with the substrate. The interface bonding zone is a thick planar crystal, which gradually develops into columnar crystal and dendritic crystal of the cladding layer. The microstructure of coating mainly depends on the ratio value of temperature gradient *G* to the solidification rate *R* [23]. There is a large temperature gradient at the interface between the substrate and the molten pool, the solidification rate *R* tends to zero, this makes the *G/R* value tends to infinity. Therefore, the microstructure grows epitaxy as a planar crystalline from the substrate. As the solidification proceeds, the temperature gradient decreases due to the decreasing molten pool temperature, leading to the dropping of the *G/R* value. The microstructure changes from planar crystal to columnar crystal. The dendritic crystals are formed in the middle and upper part of the coating with a decrease of the *G/R* value, as shown in Figure 3c.

**Figure 4:**
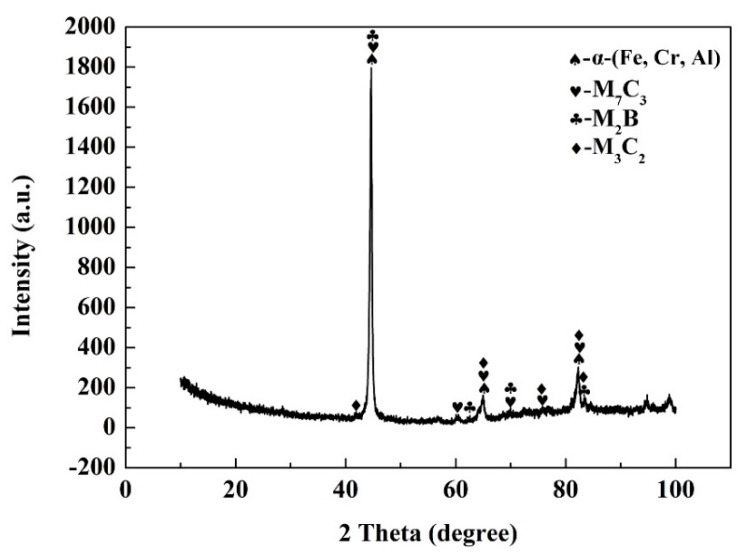
The X-ray pattern of high aluminum and chromium Fe-B-C coating.

Figure 4 presents the X-ray pattern results of the coating. The matrix occupies the peak location (2θ = 44.49°, 64.31°, and 81.97°) of the α-Fe, which is slightly less than the peak values (2θ = 44.69°, 64.96°, and 82.36°) of α-Fe in 2Cr13 steel [24]. The compositions of various phases were detected by EPMA, as shown in Table 2. It can be seen that the elements Cr, Al, etc. are dissolved into the α-Fe. Moreover, the atomic radii of Fe and Cr are 118 and 117 pm respectively, which is very helpful for Cr to be solidly dissolved in α-Fe, resulting in lattice mismatches, leading to a left shift of 2θ [25]. The Al and Cr elements dissolve into the matrix to form α-(Fe, Cr, Al) solid solution. The eutectic microstructure is mainly composed of M_2_B borides and M_3_C_2_ and M_7_C_3_ carbides, M mainly represents the elements of Fe, Cr, etc. [26]. The contents of Cr, B, and C in the eutectic phase zone 2 and zone 3 are much higher than that in dendrite (zone 1), while the contents of Fe, Al, and Si are higher in dendrite. This indicates that Cr element is easy to form boroncarbides among dendrites, while Al element are easier to be solidly dissolved into matrix to form matrix α-(Fe, Cr, Al) solid solution, which is conducive to improving the hardness and wear resistance of the coating.

**Figure 5:**
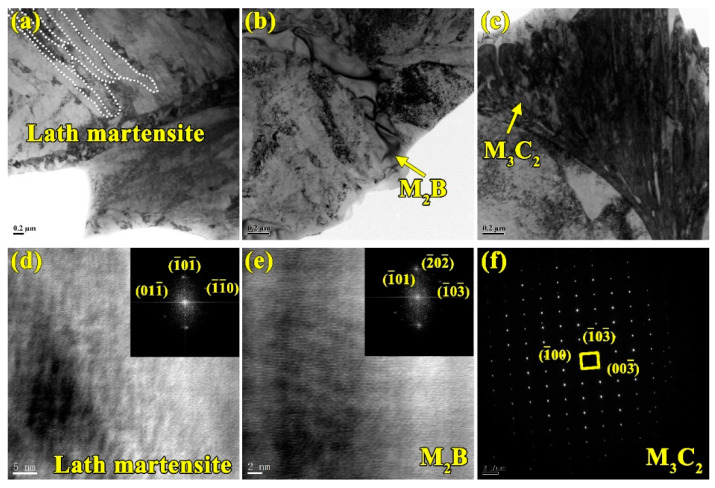
Transmission Electron Microscopy (TEM) of high aluminum and chromium Fe-B-C coating: (**a**–**c**) bright field images; (**d**) high resolution image of lath martensite and corresponding selective area diffraction (SAD) pattern; (**e**) high resolution image of M_2_B and corresponding SAD pattern; and (**f**) the SAD pattern of M_3_C_2_.

The TEM tests are conducted to further determine the type of matrix and interdendritic boroncarbides in coating, as shown in Figure 5. From Figure 5a,d, the matrix of the coating is identified as the lath martensite with face-centered-cubic (fcc) structure. The large-sized block phase formed between dendrites are orthorhombic M_2_B, and the small rod-shaped phase between the block M_2_B are M_3_C_2_ with orthogonal structure and M_7_C_3_ with orthorhombic structure carbides. This network structure of eutectic structure is very similar to the microstructure of the cast Fe-Cr-B-Al alloy [27]. The size and distribution of the M_2_B borides, M_3_C_2_ and M_7_C_3_ carbides in coating mostly depend on the microstructure evolution mechanism from the initial reactant powders. It is very interesting to study the microstructure evolution mechanism during the laser cladding. The powders dissolve into atoms in the molten pool under the laser beam, the Cr_2_B phase firstly precipitates from the molten pool during the solidification according to the results in Figure 2. From Figure 3d, it can be speculated that as solidification process progresses, the remaining atoms begin to nucleate and grow on the Cr_2_B and the M_7_C_3_ and M_3_C_2_ precipitates.

### 3.3. Microhardness

**Figure 6:**
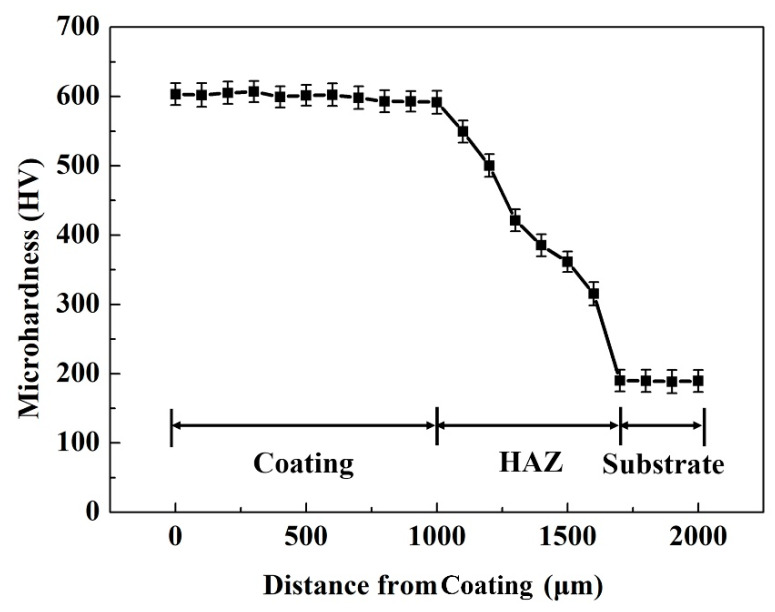
The microhardness profile along the cross-section of the coating.

Figure 6 shows the microhardness profile along the cross-section of the coating. It can be seen that the microhardness exhibits a gradient variation from the substrate to the coating, gradually increases from the interface bonding zone, heat-affected zone to the coating, which is mainly connected with microstructure evolution of the coating. According the results of microstructure analysis, the microstructure gradually transforms columnar crystals to equiaxed grains from the substrate to the coating. H. Xie et al. reported the hardness of the equiaxed grains is much higher than that of the columnar grains [28]. J. Li et al. thought the heat-affected zone (HAZ), as a transition zone, can effectively decrease the stress concentration and enhance the interface bonding [29]. It also can be seen that the microhardness of the coating has no major fluctuation and reaches 620 HV, which is 3.3 times that (190 HV) of the substrate. This indicates the microstructure is very evenly distributed and no obvious cracks and holes defects were produced. According to the microstructure analysis, the matrix of the coating is the α-(Fe, Cr, Al) solid solution martensite. The Al and Cr elements plays a solid solution strengthening role. The microhardness of martensite (480–560 HV) is higher than that of the ferrite (160–220 HV) [30]. Furthermore, the M_2_B borides, M_3_C_2_ and M_7_C_3_ carbides with high hardness precipitates in the coating. Z.F. Huang found the microhardness of Fe_2_B is about 1500 HV [31], furthermore, J. Lentz found the hardness of the M_2_B is lightly higher than that of the Fe_2_B [32,33]. The hardness of M_7_C_3_ is reported to be about 1800 HV by Tassin [34]. H.G. Fu et al. found the hardness of the M_3_C_2_ is slightly lower than that of the Fe_2_B about 1300 HV [35]. The martensitically hardened Fe matrix and the high hardness of the hard phases lead to an improvement of microhardness of the coating.

### 3.4. Wear Resistance

**Figure 7:**
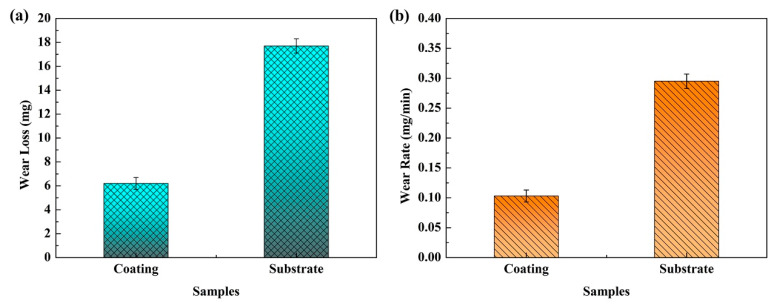
The wear loss (**a**) and wear rate (**b**) of the tested samples.

Figure 7 shows the wear loss and wear rate of the substrate and coating. As can be seen from Figure 7a, the wear loss of substrate reaches 17.7 mg, while that of the coating is only 6.2 mg. According to Equation (1), the calculated wear rates of the coating and substrate are 0.295 mg/min and 0.103 mg/min, respectively, as shown in Figure 7b. The wear resistance of laser cladded coating is ~2-times higher than that of substrate. H. Berns studied the effect of B/C on wear resistance of the as-cast Fe-B-C alloy, and found the as-cast Fe-B-C alloy exhibited excellent wear resistance due to the main Fe_3_(C,B) phase with high hardness (1300 ± 50 HV) when the B/C was 1/3 [36]. According to the microstructure analysis, the Al and Cr elements dissolve into the matrix to form α-(Fe, Cr, Al) solid solution, furthermore, the M_2_B borides and M_7_C_3_ and M_3_C_2_ carbides of the coating have high hardness, which is higher than the Fe_3_(B,C) phase, exhibiting better wear resistance. The high hardness borides and carbides can inhibit the wear of the grinding ball on the matrix, while the matrix with high hardness can also prevent the boron-carbons from falling off. The boroncarbide network structure in the coating makes it more stable, while the M_23_C_6_ carbides in the substrate are mainly distributed along grain boundaries, and the microhardness of α-Fe matrix is lower than that of the martensite. After the application of the load, the M_23_C_6_ carbides with high hardness may be pressed into the matrix, resulting in severe cutting to the matrix. The reciprocating wear can further aggravate the wear of the substrate.

**Figure 8:**
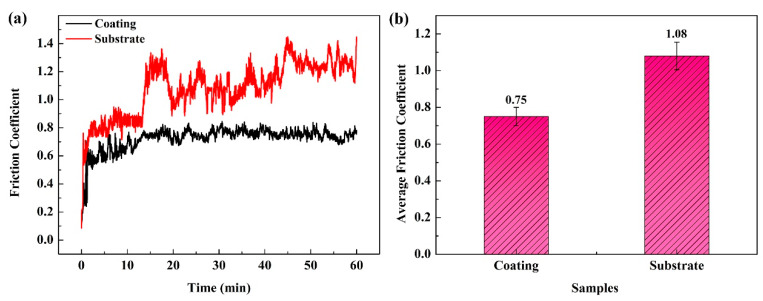
The variation of friction coefficient with time (**a**) and average friction coefficient (**b**) of the tested samples.

Figure 8 presents the variation of friction coefficient with time and average friction coefficient of the tested samples. It can be seen that the friction coefficient of the substrate increases quickly and remain stable in the first 12 min, and exhibits large fluctuation in the subsequent wear process which is caused by the incomplete occlusal of samples and grinding balls. Comparing with the friction coefficient of the substrate, the fluctuation of friction coefficient of the coating decreases significantly, as shown in Figure 8a. From Figure 8b, it is clear that the average friction coefficient of the coating is 0.75, which is much lower than that of the substrate (1.08).

### 3.5. Wear Mechanisms

**Figure 9:**
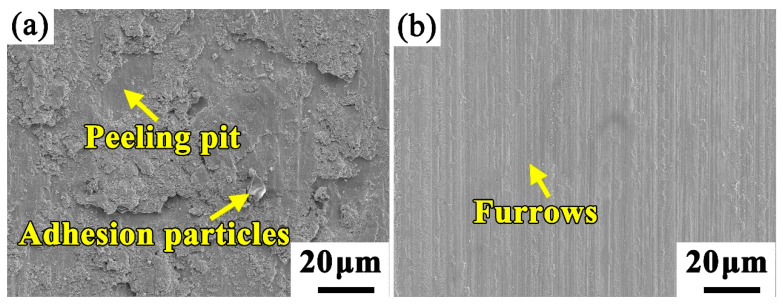
The morphologies of the worn surface of (**a**) substrate and (**b**) high aluminum and chromium Fe-B-C coating.

Figure 9 shows the morphologies of the worn surface of the tested samples. It can be seen that large pits and abrasive particles can be observed on the worn surface of the substrate, as shown in Figure 9a, which indicates the wear mechanism of the substrate is composed of the adhesive wear and abrasive wear. However, the worn surface of the coating shows a completely different morphology. A lot of shallow furrows appear on the worn surface, and contains some abrasive particles with small size. The formation of the abrasive particles causes the three body abrasion, which may be the main reason for forming the furrows. This suggests the wear mechanism of the coating is mainly comprised of slight abrasive wear.

The wear resistance of materials is mainly related to morphology and microstructure [37]. The substrate is composed of soft α-Fe matrix and little hard M_23_C_6_ carbides distributed along the grain boundary [38]. The soft matrix does not provide adequate protection for hard M_23_C_6_ carbides, resulting in the peeling off of hard carbides. This also is the main reason to bring about the fluctuation of friction coefficient of substrate. The martensite and network structure comprising of M_2_B borides and M_7_C_3_ and M_3_C_2_ carbides are observed in the coating, as shown in Figure 5c,d. The martensite is stiffer than α-Fe ferrite, which suppresses the falling off of the hard phase. Moreover, the hard phases such as M_2_B borides and M_7_C_3_ and M_3_C_2_ carbides exhibit network structure. Compared with granular and block M_23_C_6_ carbides in the substrate, these continuous network structures are more resistant to abrasion, which can protect the martensitic matrix, thus the wear resistance of the coating is improved.

## 4. Conclusions

A novel high aluminum and chromium Fe-B-C coating which compositions are designed using the Thermo-Calc^®^ software was fabricated using laser cladding technology. The microstructure, microhardness and wear resistance were systematically investigated. The main conclusions of this research can be drawn as follows:

1. The coating is dense and has good metallurgical bond with the substrate. The microstructure is mainly composed of α-(Fe, Cr, Al) lath martensite, orthorhombic M_2_B borides, orthogonal M_3_C_2_, and orthorhombic M_7_C_3_ carbides.
2. The microhardness of the coating can reach to 620 HV which is 3.3-times higher than that of the substrate (190 HV). This is mainly due to the formation α-(Fe, Cr, Al) lath martensite, M_2_B borides, and M_3_C_2_ and M_7_C_3_ carbides with high hardness.
3. The coating shows lower friction coefficient of 0.75 than that of the substrate (1.08). The wear rate of the substrate and the coating are 0.295 and 0.103 mg/min, respectively, indicating the coating exhibits excellent wear resistance. The wear mechanism transforms severe adhesive wear and abrasive wear of the substrate to slight abrasive wear of the coating.

## Figures and Tables

**Table 1:** The chemical compositions of the high aluminum and chromium Fe-B-C cladding powder (wt.%).

Elements	Al	Cr	B	Si	Mn	C	Fe
Content	6.0	10.0	1.5	0.6	0.7	0.3	Bal.

**Table 2:** Chemical compositions of the phases with different morphologies in high aluminum and chromium Fe-B-C coating (wt.%).

Content	Fe	Cr	B	Al	Si	Mn	C
Zone1	84.175	12.271	0.44	2.271	0.494	0.628	0.554
Zone2	80.647	13.756	0.926	1.808	0.465	0.664	1.259
Zone3	70.813	21.483	3.623	0.692	0.193	0.837	3.105
